# Meeting report ‘Microbiology 2023: from single cell to microbiome and host’, an international interacademy conference in Würzburg

**DOI:** 10.1093/femsml/uqae008

**Published:** 2024-04-05

**Authors:** Pascale Cossart, Jörg Hacker, David H Holden, Staffan Normark, Jörg Vogel

**Affiliations:** Institut Pasteur, 28 rue du dr Roux, Paris 75015, France; German National Academy of Science Leopoldina, Jägerberg 1, D-06108 Halle, Germany; Department of Infectious Disease, Centre for Bacterial Resistance Biology, Flowers Building, South Kensington Campus, Exhibition Road, Imperial College London, London SW7 2AZ, United Kingdom; Karolinska Institute, Tumor-och-cellbiologi, C1 Microbial Pathogenesis, 17177 Stockholm, Sweden; Faculty of Medicine, Institute for Molecular Infection Biology (IMIB), University of Würzburg, D-97080 Würzburg, Germany; Helmholtz Institute for RNA-based Infection Research (HIRI), Helmholtz Centre for Infection Research (HZI), Josef-Schneider-Str2/Gebaude D15; É. D-97080 Würzburg, Germany

**Keywords:** microbiome, microbiology, infection biology, host–microbe interaction, bacterial immunity, pathogen–phage interaction

## Abstract

On September 20–22 September 2023, the international conference ‘Microbiology 2023: from single cell to microbiome and host’ convened microbiologists from across the globe for a very successful symposium, showcasing cutting-edge research in the field. Invited lecturers delivered exceptional presentations covering a wide range of topics, with a major emphasis on phages and microbiomes, on the relevant bacteria within these ecosystems, and their multifaceted roles in diverse environments. Discussions also spanned the intricate analysis of fundamental bacterial processes, such as cell division, stress resistance, and interactions with phages. Organized by four renowned Academies, the German Leopoldina, the French Académie des sciences, the Royal Society UK, and the Royal Swedish Academy of Sciences, the symposium provided a dynamic platform for experts to share insights and discoveries, leaving participants inspired and eager to integrate new knowledge into their respective projects. The success of Microbiology 2023 prompted the decision to host the next quadrennial academic meeting in Sweden. This choice underscores the commitment to fostering international collaboration and advancing the frontiers of microbiological knowledge. The transition to Sweden promises to be an exciting step in the ongoing global dialogue and specific collaborations on microbiology, a field where researchers will continue to push the boundaries of knowledge, understanding, and innovation not only in health and disease but also in ecology.

## Introduction

Learned societies play important roles in disseminating the latest discoveries and developments in their respective scientific discipline and in organizing a community of scientifically like-minded people. This is also true for national academies of sciences, which strive to amplify the impact and visibility of the many different disciplines they cover. One instrument for achieving this is organizing international meetings in order to bring together thought leaders from a field of interest and enable scientific exchange between students, young faculty, and established researchers in that community. On 20–22 September 2023, the national academies of four European countries joined forces to put the spotlight on microbiology, which is one of the most thriving areas of the life sciences and remains a source of unexpected findings and new principles that later find application in biomedicine, biotechnology, and ecology, to name just a few.

The four academies involved were the German National Academy of Sciences Leopoldina, represented by Jörg Hacker and Jörg Vogel; the Académie des sciences de l’Institut de France, represented by Pascale Cossart; the British Royal Society, represented by David Holden; and the Swedish Kungl. Vetenskapsakademien, represented by Staffan Normark. It was the first time for these four countries to run a joint interacademy conference but not the first time for them to collaborate in the organization of microbiology-centric meetings. In fact, Jörg Hacker and Pascale Cossart organized a FEMS/Leopoldina Symposium ‘Emerging Topics in Microbial Pathogenesis’ at the Juliusspital in Würzburg in April 2010. This then turned into a meeting series, with the next conference entitled ‘The new microbiology’ taking place at the Institut de France, Parison 14–16 May 2012. While the Académie des sciences took the lead, both the Leopoldina and The Royal Society were involved, too (Radoshevich et al. 2012). Next, these three academies partnered to organize a conference called ‘The New Bacteriology’ at the Royal Society in London on January 28–19 January 2016 (Cossart et al. 2016). Common to all these conferences was that thy aimed to highlight new concepts and approaches that fueled the recent renaissance of microbes as exciting organisms to study.

**Figure 1.**
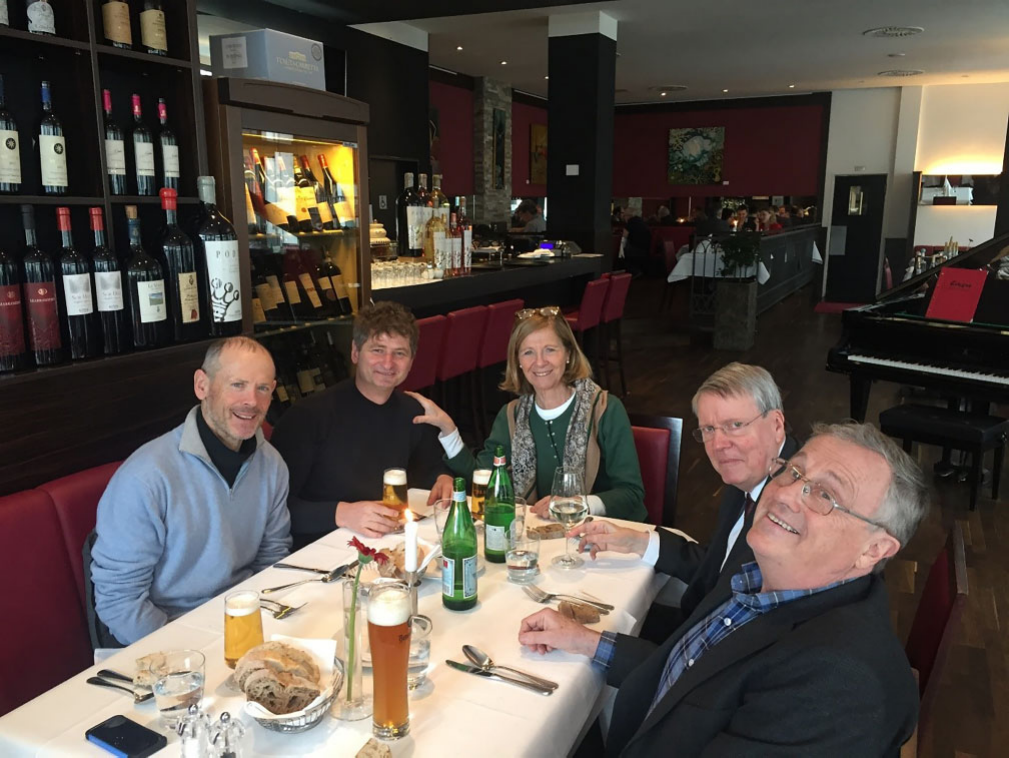
The organizers David Holden, Jörg Vogel, Pascale Cossart, Jörg Hacker, and Staffan Normark (from left to right) work-lunching in Berlin in April 2019 and having a first go at planning what back then was meant to be Microbiology 2020 and because of the COVID-19 pandemic turned out to be Microbiology 2023.

Plans were underway to hold the next meeting, for which the present organizers originally met in Berlin in April 2019 (Fig. 1), to agree on speakers and prepare the conference schedule for the year after. However, the COVID-19 pandemic starting in spring 2020 thwarted their plans and made it literally impossible to firmly fix a date for the next 2 years; while it was the organizers’ ambition to secure a list of high-profile international speakers, sometimes unpredictable travel restrictions made planning very difficult. Yet, as the skies started clearing up in early 2023, they were pleasantly surprised by the unabated commitment of the invited speakers to come and speak in September that year. This time called ‘Microbiology 2023: From single cell to microbiome and host’, the conference built on the successful format of previous meetings: starting on Wednesday afternoon and finishing Friday noon, with half-hour speaking slots for a total of 22 invited speakers.There was plenty of time and opportunity for early career researchers to present their work as part of a poster session and to mingle with the speakers as well as attending established scientist.

**Figure 2.**
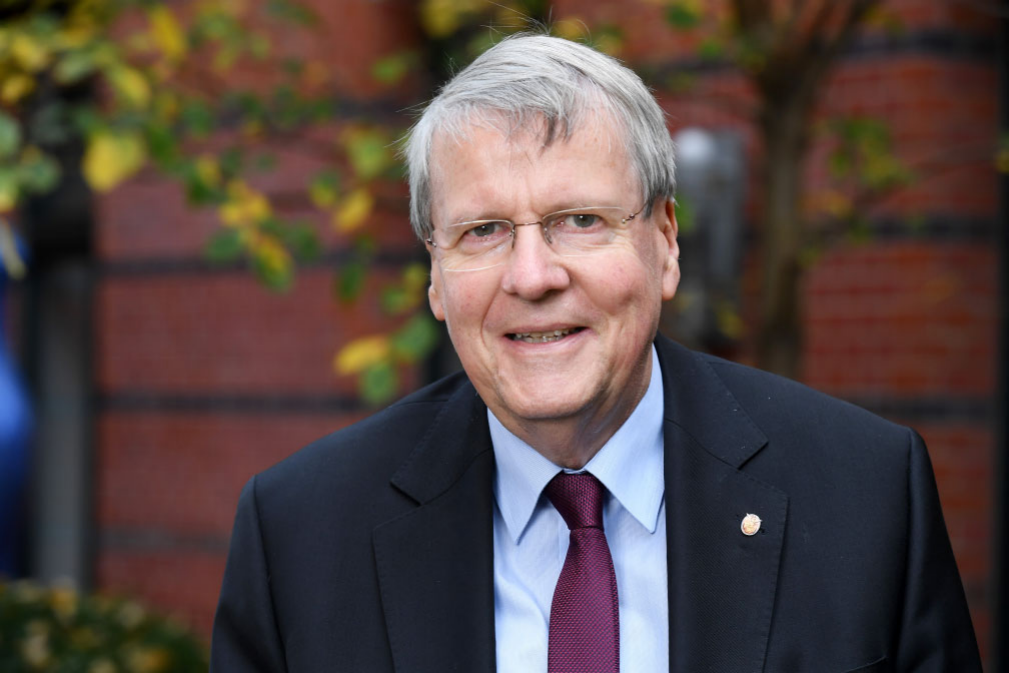
Jörg Hacker, past president of the German National Academy of Sciences (2010–2020) and founding director of the Institute for Molecular Infection Biology of the University of Würzburg (1993–2008).

**Figure 3.**
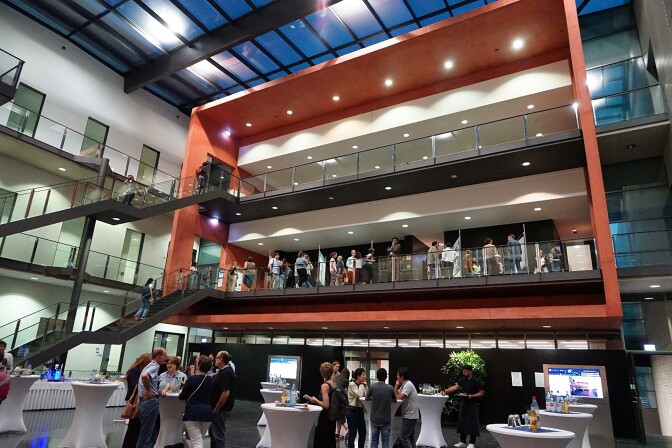
Inside the building of the Institute for Molecular Infection Biology.

Before we provide an overview of the scientific programme, we would like to mention that there was more than one reason for the conference to return to Würzburg after Paris and London, and these ultimately relate to the person of Jörg Hacker (Fig. 2). To begin with, Hacker served the Leopoldina as President for 10 years, from 2010 to 2020. With Microbiology 2023 the Leopoldina wished to recognize its outgoing President’s many contributions in the fields of microbiology, infectious diseases research, and beyond. Moreover, Würzburg boasts a vibrant community of microbiologists and infectious disease researchers with a critical mass that hardly matched by other places in Germany. Hacker was one of the founding fathers of this community, through both his own research into pathogenicity island and genomic and extrachromosomal elements of bacterial pathogens and his visionary activities as the founding director of the Institute of Molecular Infection Biology (IMIB) of the University of Würzburg (1993–2006). He also cofounded and led for many years the Würzburg Research Center for Infectious Diseases (ZINF), whose pioneering young investigator programme facilitated the early independence of dozens of young scientists from bacteriology, parasitology, and fungal biology. Although he left Würzburg almost 20 years ago to serve as the President of the federal Robert Koch Institute, before taking the helm at the Leopoldina, he continued to provide support to the Würzburg research community. His advice was crucial in a national competition for new research institutes in 2016, which resulted in the founding the Helmholtz Institute for RNA-based Infection Research (HIRI), Würzburg’s first federally funded research institute in the life sciences. Moreover, while still in Würzburg, he helped to secure the funds for a new research building which now hosts the IMIB and provided the venue for Microbiology 2023 and its ∼300 participants (Fig. 3).

## Scientific programme

The conference commenced with an opening presentation by Jörg Vogel in his capacity of the local host and director of the IMIB and the HIRI. He introduced his fellow organizers as well as additional invited sessions chairs: Thomas Rudel, Chase Beisel, and Cynthia Sharma (all Würzburg), Franz Narberhaus (Bochum) and Birgitta Henriques Normark (Stockholm). There were four sessions in total, the first on Wednesday, two Thursday morning and afternoon, and the last on Friday morning.

### Session 1: infection biology and microbiome (Fig. 4)

#### Internal conflicts in *Salmonella* persisters

Sophie Helaine (Harvard Medical School, Boston, USA) discussed bacterial persistence and how during infection, *Salmonella* respond to engulfment by macrophages by forming many persisters (Helaine et al. 2014). These persisters escape the combined action of the antibiotic and host immune defence by adopting a nongrowing state. Sophie presented unpublished data (Molly Sargen and Sophie Helaine, unpublished) showing how DNA damage occurring in persisters while within macrophages (Hill et al. 2021) induces prophage reactivation in these bacteria that nonetheless avoid lysis. She presented that Prophage Competition Elements protect persisters and provided molecular details of activity of RemAIN, one such Prophage Competition Element.

**Figure 4.**
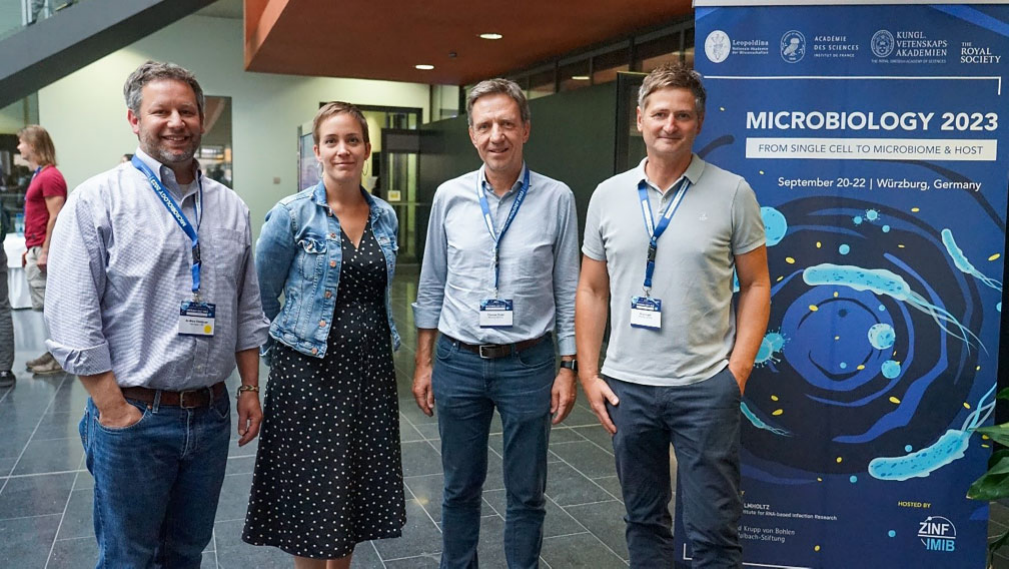
From left to right the speakers of the ‘Infection biology and microbiome’ session Andrew Goodmann and Sophie Helaine as well as session chairs Thomas Rudel and Jörg Vogel. The two other speakers, not pictured here, were Susan Bullman and Andreas Bäumler.

#### The role of intratumoural microbiota within the tumour environment: from niches to single cells

Susan Bullman (Fred Hutchinson Cancer Center, Seatle, WA, USA) discussed how the tumor microenvironment (TME), consisting of a complex network of malignant and nonmalignant cells, significantly impacts tumour behaviour. Molecular analyses have revealed an intratumoural microbiota within human solid cancers, yet understanding their interactions with human tumour components and contributions to disease progression is limited (Bullman 2023). Recent findings from the Bullman’s lab uncover how intratumoural bacterial communities contribute to cancer progression in oral and colorectal cancers (Galeano Niño et al. 2022). Using spatial transcriptomics, spatial proteomics, and single-cell sequencing (INVADEseq; Galeano Niño et al. 2023), the research reveals intratumoural bacteria localize to immunosuppressive tumour regions and infect cancer epithelial cells, upregulating cancer-related pathways. These insights offer promise for targeted therapies disrupting bacterial–human interactions in the TME.

#### Host–microbe interactions underlying colonization resistance

Andreas Bäumler (UC Davis, USA) described that antibiotic prophylaxis sets the stage for an intestinal *Candida albicans* bloom (Pappas et al. 2018), but that the resources driving this expansion are incompletely understood. Butyrate-producing *Clostridia* species maintain epithelial hypoxia but are depleted by antibiotic treatment (Byndloss et al. 2017). Notably, increased epithelial oxygenation after *Clostridia* depletion drove a postantibiotic *C. albicans* expansion. Commensal *Clostridia* species could be replaced functionally with 5-aminosalicylic acid (5-ASA), which activated mitochondrial oxygen consumption to restore epithelial hypoxia and reinstate colonization resistance against *C. albicans* (Savage et al. 2023). Thus, 5-ASA treatment is a nonbiotic intervention that restores colonization resistance against *C. albicans*.

#### Microbiome dynamics during infection

Andrew Goodman (Yale School of Medicine, USA) presented recent work characterizing interbacterial interactions in the gut microbiome. The seminar focused on contact-dependent antagonism, highlighting examples *in vitro*, in animal models, and inferences from human metagenomic data that each illustrate the role of these interactions in shaping gut microbiome composition (Wexler et al. 2016, Verster et al. 2017). Andy then presented ongoing studies aimed at dissecting the mechanism and impact of specific antibacterial toxins produced by commensal microbes during health and disease. These experiments combine gnotobiotics, synthetic biology (Lim et al. 2017), biochemical, and structural approaches to understand the mechanisms of interbacterial antagonism in the gut microbiome.

### Session 2: phage biology and defence (Fig. 5)

#### Deciphering the immune system of bacteria

Rotem Sorek (Weizmann Institute, Rehovot, Israel) presented data on mechanisms of bacterial defence against phages. He showed how such systems can be identified by systematic analysis of available microbial genomes, an approach that recently led to the discovery of many dozens of new defence systems (Doron et al. 2018). These analyses currently transform our understanding of bacterial immunity, and surprisingly show that key components of the human innate immune system evolved from bacterial defence against phages (Wein and Sorek 2022). Sorek showed how studying bacterial immune system can reveal new concepts in animal and plant innate immunity (Rousset et al. 2023).

**Figure 5.**
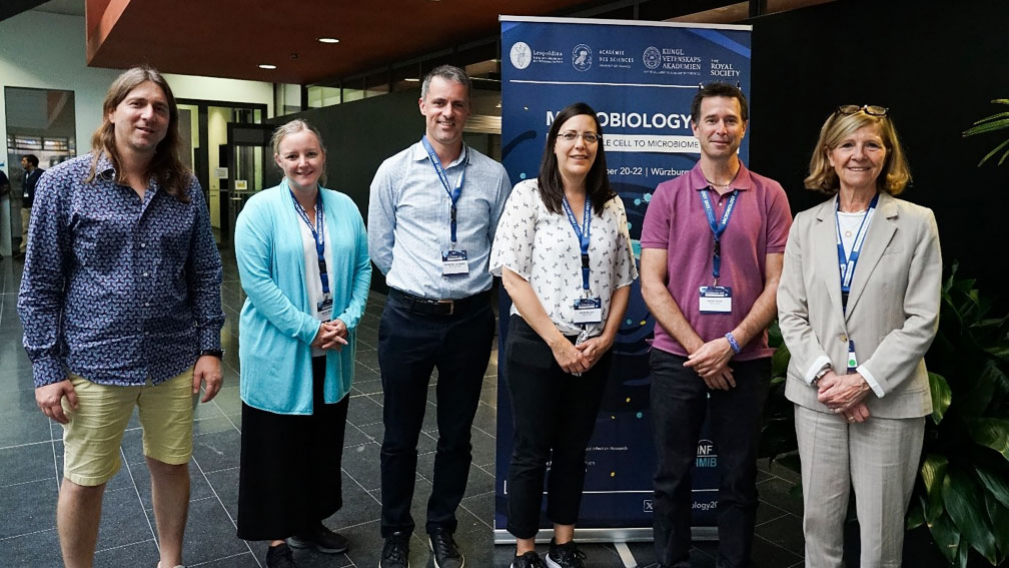
From left to right the speakers of the ‘Phage biology and defence’ session Nassos Typas, Melanie Blokesch, Joseph Bondy-Denomy, Karen Maxwell, and Rotem Sorek, as well session chair Pascale Cossart. The other chair, Chase Beisel, is not in the picture.

#### What to do when CRISPR fails? Back-up plans for phage defence

Joseph Bondy-Denomy (University of California San Francisco, USA) described how bacteriophages antagonize the cyclic-oligonucleotide based antiphage signaling system (CBASS) using an anti-CBASS protein (Acb2). Acb2 sequesters or ‘sponges’ up the signaling nucleotides that cyclase enzymes generate in response to phage infection (Huiting et al. 2023). Acb2 acts as a hexamer, which generates three cyclic dinucleotide (CDN) binding sites. Acb2 is encoded by many phages and prophages across Gram-negative bacteria and recent work has revealed a second binding site that is distal from the CDN binding site, which binds with high affinity to cAAA and cAAG CBASS signalling trinucleotides (Cao et al. 2024). Together, the ∼90 amino acid Acb2 protein likely inactivates nearly all CBASS systems.

#### Inhibition of virion assembly in antiviral defence

Karen Maxwell (University of Toronto, Toronto, Canada) described a new antiphage defence system encoded by a *Pseudomonas aeruginosa* prophage. This protein inhibits virion assembly of superinfecting phages through an interaction with the tape measure protein, a critical component of long-tailed phages. Prophages that have this defence encode a counter-defence protein that is expressed during the lytic cycle that allows them to evade the assembly inhibition. As prophage survival depends on survival of the bacterial host, they frequently encode defence systems to protect the cell in which they reside (Bondy-Denomy et al. 2016, Rocha and Bikard 2022, Patel and Maxwell 2023); understanding these defences and how they protect their bacterial host will provide critical insight into the phage-host evolutionary arms race.

#### Enormous diversity of antiretron proteins in phages

Nassos Typas (EMBL Heidelberg, Germany) first discussed that Restriction-modification systems and CRISPR, tools that have propelled genetic engineering, are systems that bacteria use to defend phage. Yet, only recently have we started to understand the multitude of systems bacterial employ in this warfare. Among them, bacterial retrons, containing the first prokaryotic reverse transcriptases discovered, were recently shown to act as phage defence systems (Gao et al. 2020, Millman et al. 2020, Bobonis et al. 2022). To explore on possible ways phages use to bypass retrons, Nassos Typas presented a screen his lab performed, using metagenomic phage DNA libraries from diverse environments, to identify proteins that inhibit the effector of one such retron, Retron-Sen2. This way, they identified dozens of small phage proteins that block the effector toxicity. The majority shared no obvious sequence homology with known systems or between each other, and many inhibited divergent retrons, also during phage infection. By combining genetics, biochemistry, and structure prediction, he presented evidence of convergent evolution in the way blockers counteract the defence system.

#### Molecular secrets of seventh pandemic *Vibrio cholerae*

Melanie Blokesch (EPFL, Switzerland) presented new findings on the defence mechanisms of *Vibrio cholerae*. Her focus was on the specific strains responsible for the ongoing seventh cholera pandemic, also recognized as the seventh pandemic O1 El Tor (7PET) clade (Mutreja et al. 2011). Within this lineage, recent research has highlighted several defence systems primarily encoded within long-known but previously understudied pathogenicity islands/mobile genetic elements (Dziejman et al. 2002). These systems include two DNA defence modules (Ddm), which play a critical role in safeguarding *V. cholerae* from phages and/or plasmids (Jaskólska et al. 2022). Further investigations are needed to comprehend how these newfound defence systems contribute to the success of the 7PET clade of *V. cholerae*.

### Session 3: molecular biology and bacterial cell biology (Fig. 6)

#### Dynamics of the peptidoglycan synthesis machinery during *Staphylococcus aureus* cell division

Mariana G. Pinho (ITQB, Universidade Nova de Lisboa) explored FtsZ treadmilling’s role in bacterial cell wall synthesis and division, focusing on *Staphylococcus aureus*, a deadly antibiotic-resistant pathogen. FtsZ treadmilling involves asymmetric FtsZ filament polymerization at the plus end and depolymerization at the minus end, causing FtsZ polymer movement around the division site. Mariana showed that FtsZ treadmilling is crucial only during early cytokinesis stages but becomes dispensable afterwards (Monteiro et al. 2018). Initial hypotheses suggested its role in promoting directional movement of septal peptidoglycan synthases around the division site (Bisson-Filho et al. 2017, Yang et al. 2017). However, single molecule tracking microscopy showed that velocity of FtsW/PBP1, staphylococcal essential septal peptidoglycan synthases, remains unaffected upon FtsZ treadmilling inhibition. In contrast, peptidoglycan synthesis inhibition significantly hindered septum constriction and FtsW/PBP1 movement (Schäper et al. 2023), emphasizing peptidoglycan synthesis’ critical role in driving cytokinesis.

**Figure 6.**
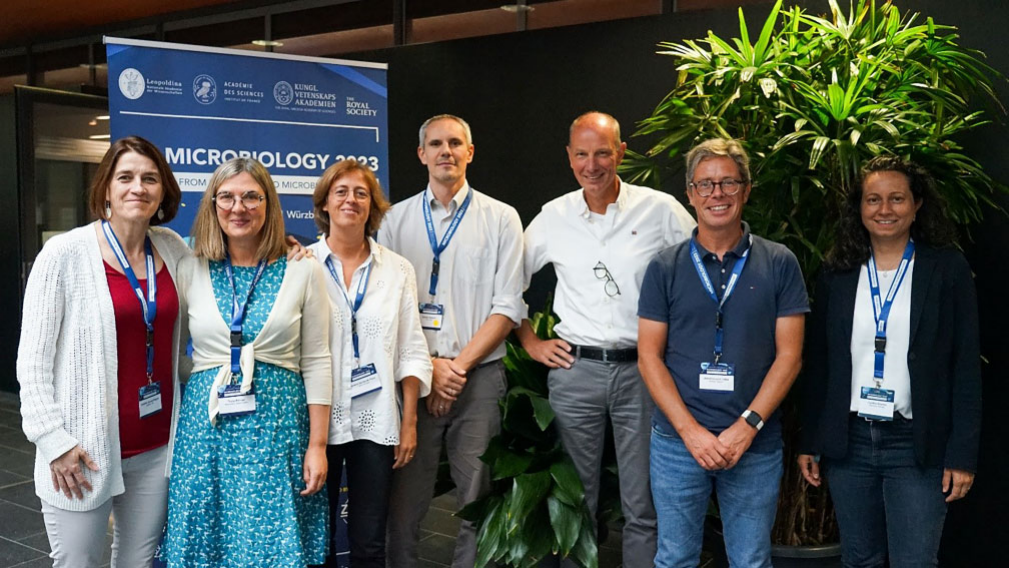
From left to right the speakers of the ‘Molecular biology and bacterial cell biology’ session Christine Jacobs-Wagner, Tracy Palmer, Mariana G. Pinho, Davide Sassera, and Jean-François Collet (second to the right). Session chairs were Franz Narberhaus (third to the right) and Cynthia Sharma (to the right).

#### Chromosome segregation : how bacteria do more with less

Christine Jacobs-Wagner (Howard Hughes Medical Institute, Stanford University, Stanford, USA) presented unpublished work that addressed the long-standing mystery surrounding the mechanism of chromosome segregation in the model bacterium *Escherichia coli*. With the support of single-cell quantitative imaging data across various nutrient conditions, she expanded on a recent study from her group on chromosome folding (Xiang et al. 2021) to suggest that excluded volume effects involved in chromosome compaction also result in effective displacement forces that contribute to the demixing and segregation of sister chromosomes.

#### Novel effector proteins of the *S. aureus* Type VII secretion system

Tracy Palmer (Biosciences Institute, Newcastlle University, Newcastle, England) discussed targeting of antibacterial toxins to the type VII secretion system in *S. aureus* (Cao et al. 2016). She presented data showing that three small helical proteins, EsxB, EsxC, and EsxD are required for export of the EsaD nuclease toxin (Yang et al. 2023). These proteins bind to EsaD’s N-terminal LXG-like domain resulting in formation of a long helical stalk structure. She also described TslA, the first example of a T7SS toxin with a reverse domain arrangement (Garrett et al. 2023). Biochemical evidence was presented demonstrating that it has phospholipase activity and that it is toxic to bacteria lacking TilA immunity proteins.

#### Mechanical interplay of the OM (outer membrane) and the peptidoglycan layer in Gram-negative bacteria: a key to resisting turgor pressure and cell bursting

Jean-François Collet (UC Louvain, Belgium) in his presentation, first revisited the understanding of how Gram-negative bacteria handle osmotic challenges. Traditionally, it was believed that peptidoglycan provided the mechanical support to resist increased turgor pressure during hypoosmotic stress (Egan et al. 2020). However, Collet presented a combination of genetics, single-cell fluorescence microscopy, and biochemistry data that challenged this notion. He highlighted that while peptidoglycan is essential, it is not sufficient for survival under osmotic stress. Collet proposed that attaching peptidoglycan to the outer membrane forms a mechanical unit that allows periplasmic pressure buildup, thus balancing turgor pressure across the inner membrane and preventing cell lysis (Asmar et al. 2017, Deghelt et al. 2023).

#### Intramitochondrial localization of *Midichloria* bacteria: where, when and how

Davide Sassera (University of Pavia, Italy) described advances in research on *Midichloria*, a genus of tick-borne mutualist bacteria, found to reside within the intermembrane space of different tick species (Stavru et al. 2020). Advanced 2D and 3D electron microscopy suggested that *Midichloria* bacteria influence mitochondrial network fragmentation (Uzum et al. 2023). Additionally, comparative genomics revealed a possible connection between the bacterium’s ability to colonize mitochondria and the presence of specific genetic traits, including type IV secretion system and flagellum (Floriano et al. 2023). Current analyses are focused on investigating protein–protein interactions between *Midichloria* and the tick, looking for the molecular mechanisms at the basis of this unique symbiosis.

### Session 4: molecular infection biology (Fig. 7)

#### The cytosolic LPS-sensing noncanonical inflammasome pathway in bacterial infection

Feng Shao (National Institute of Biological Sciences, Beijing) reported the latest development about the cytosolic LPS-sensing caspase-11/4 noncanonical inflammasome. He described in detail how caspase-11/4 recognize LPS and becomes activated (Shi et al. 2014). The activated caspases, specifically the p20/p10 autoprocessed form, use an exosite to recognize Gasdermin D (GSDMD) C-terminal domain, rendering a tetrapeptide-independent cleavage to liberate the pore-forming domain of GSDMD (Wang et al. 2020). He showed that this substrate-recognition mechanism also applies to caspase-1. His talk mainly covered a new finding that LPS-activated caspase-4 but not caspase-11 efficiently processes pro-IL-18 for maturation, linking infection-induced pyroptosis to antibacterial adaptive immunity (Shi et al. 2023). Structurally, caspase-4 uses the same exosite to recruit pro-IL-18. Lastly, he discussed how *S. flexneri* employs its T3SS effectors (IpaH9.8, OspC3, and IpaH7.8) to block the noncanonical inflammasome at different steps, highlighting its crucial function in antibacterial defences.

**Figure 7.**
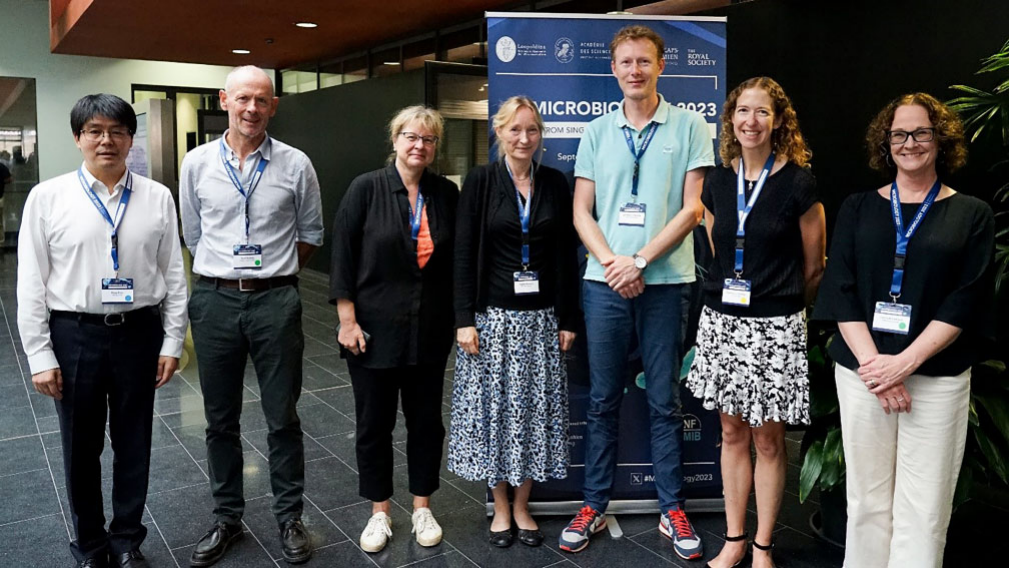
From left to right the speakers or session chairs of the ‘Molecular infection biology’ session Feng Shao, David Holden (chair), Petra Dersch, Birgitta Henriques Normark (chair), Jan-Willem Veening, Christina Stallings, and Elizabeth Hartland. Further speaker Anat Herskovits is not in the picture.

#### Legionella and the host epitranscriptome

Elizabeth Hartland (Hudson Institute of Medical Research, Australia) described how the intracellular bacterial pathogen *Legionella pneumophila* caused the post-transcriptional decay of host mRNA encoding glycolytic enzymes, thereby reducing the glycolytic activity of infected host cells. Studies of genome deletion mutants (Wibawa et al. 2022) revealed a single Dot/Icm effector protein that drove host mRNA degradation. Using iCLIP2 protocols, the bacterial effector was shown to be a new kind of host mRNA binding protein that recognized a guanine rich motif in target mRNAs (McCaffrey et al., unpublished work). This raises the possibility that *L. pneumophila* and other pathogens directly target the host epitranscriptome during infection.

#### Pathogen phage cooperation during mammalian infection

Anat Herskovits (Tel Aviv University, Israel) reported a remarkable adaptation of prophages to their pathogenic host. Prophages that inhabit pathogenic bacteria adapt their responses and regulatory machineries to support the survival of their hosts in the mammalian environment. The intracellular bacterial pathogen *Listeria monocytogenes* strain 10403S carries two phage elements in its genome, one is an active prophage producing virions, whereas the other defective producing tailocins (Argov et al. 2019). The two elements hold the capacity to trigger cell lysis upon stress, and yet during *Lm* infection of macrophage cells their response is coordinated and further adapted to avoid the production of virion/tailocin particles and cell lysis. The two elements are coregulated by the same antirepressor, encoded by the tailocin element, which triggers their induction both under stress conditions and in the mammalian niche (Argov et al. 2019). In the mammalian environment the two elements do not proceed into lytic production, yet instead conjointly mediate the excision of the prophage from the bacterial *comK* gene, a gene that was previously shown to promote *Lm*’s intracellular growth (Rabinovich et al. 2012, Pasechnek et al. 2020). Anat further demonstrated that the prophage has acquired a specific adaptation that attenuates its induction in the mammalian environment, thereby supporting *Lm*’s survival and growth. The presented data support the premise that prophages that associate with bacterial pathogens have evolved to align their response with the pathogenic lifestyle of their host, being patho-adapted to the mammalian environment.

#### Uncovering mechanisms of pneumococcal–host interactions with synthetic gene regulatory networks

Jan-Willem Veening (University of Lausanne, Switzerland) introduced phenotypic variation, where clonal bacteria exhibit different traits under identical conditions (Smits et al. 2006). This phenomenon, widespread yet poorly understood, was explored using synthetic biology and the pneumococcal capsule as an example. Veening likened pneumococcus to a cloaked Klingon Bird of Prey, hidden from scanners (or the host immune system) due to its capsule. Decloaking (losing the capsule) allows it to attack, akin to the Bird of Prey firing its phasers (and pneumococcus attaching to epithelial cells). Using CRISPRi-based synthetic gene regulatory networks, capsule production was uncoupled from its natural expression. This demonstrated that strains with oscillatory capsule production were more adept at colonizing mice than homogeneously expressing bacteria, proving the significance of gene expression heterogeneity during infection (Rueff et al. 2023).

#### Shaping *Yersinia* virulence for acute and persistent infections

Petra Dersch (University Münster, Germany) reported how the enteropathogenic pathogen *Yersinia pseudotuberculosis* is able to cause persistent infections. Using an oral mouse infection model, it was shown that the loss of the cytotoxic necrotizing factor (CNF_Y_), which activates Rho GTPases (Chaoprasid and Dersch 2021) and triggers the induction of acute inflammatory responses and tissue destruction, is sufficient to trigger a switch from acute into persistent infection (Heine et al. 2018, Chaoprasid et al. 2021). During infection, CNF_Y_ is important for the initial entry into lymphatic tissues and later for the inactivation of neutrophils, indicating that delayed colonization and a reduction of neutrophil death are crucial for the development of *Yersinia* persistence.

#### Inflammation as a driver of Mycobacterium tuberculosis pathogenesis

Christina Stallings (Washington University School of Medicine, USA) discussed the effects of inflammation on *Mycobacterium tuberculosis* pathogenesis. In particular, neutrophil recruitment and accumulation in the lungs is associated with tuberculosis disease progression, however, whether the neutrophils actively affect *M. tuberculosis* pathogenesis was unknown. Using genetic mouse models of *M. tuberculosis* infection, it was shown that neutrophils actively promote *M. tuberculosis* replication and pathogenesis (Kimmey et al. 2015). In response to *M. tuberculosis* infection, type I interferon is induced in neutrophils, which promotes the release of neutrophils extracellular traps (NETs). NETs contribute to increased pathogen replication (Sur Chowdhury et al. 2022), where inhibition of NETosis leads to better control of *M. tuberculosis* pathogenesis. Furthermore, the autophagy-associated protein ATG5 was identified as a critical regulator of type I interferon-induced NETosis during *M. tuberculosis* infection (Kinsella et al. 2023), revealing new potential targets for modulating neutrophil behaviour during *M. tuberculosis* infection to better control infection.

#### Meet-the-editor session and panel discussion with international guests

**Figure 8.**
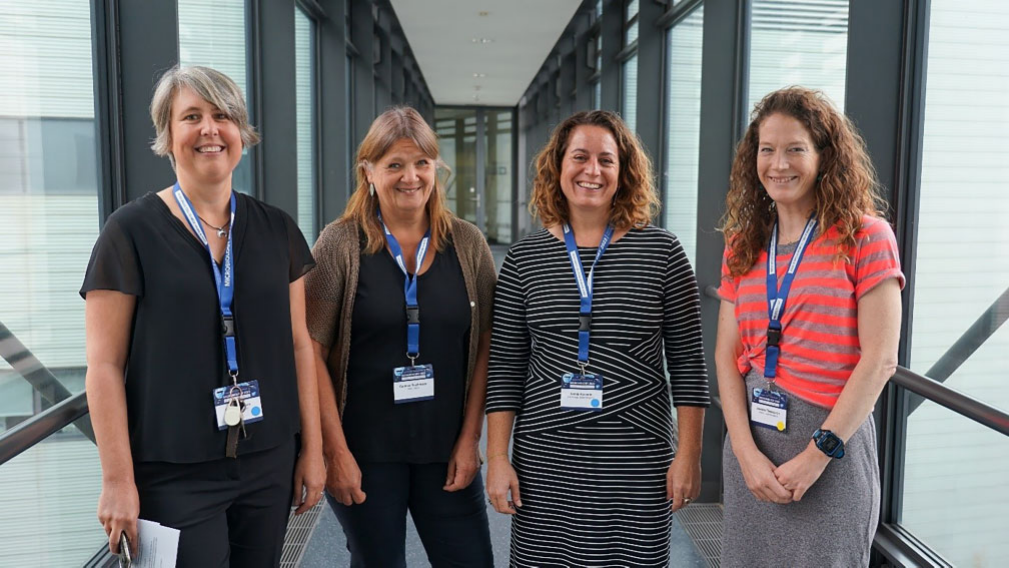
From left to right are the panelists of the ‘Meet-the-editor’ session Anke Sparmann (session chair, HIRI Würzburg), Carmen Buchrieser (*microLife*), Nonia Pariente (*PLoS Biology*), and Jessica Thompson (*Nature Microbiology*).

**Figure 9.**
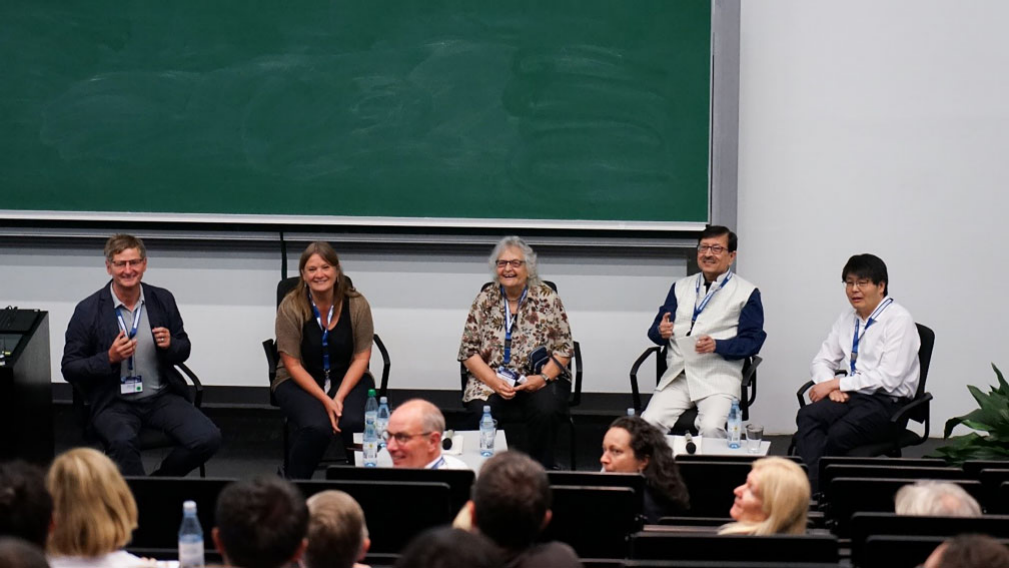
From left to right are the panelists of ‘The importance of microbiology and infectious disease research in the year 2023’ session, starting with chairs Jörg Vogel and Carmen Buchrieser, and participants Eliora Ron (Israel), Seyed E. Hasnain (India), and Feng Shao (China).

The scientific programme was complemented by two additional items on the agenda. First, an editor session (Fig. 8) chaired by Anke Sparmann, a former editor now working as a scientific writer at the Helmholtz Institute for RNA-Based Infection Research Würzburg. She hosted Nonia Pariente, editor-in-chief of *PLoS Biology*; Carmen Buchrieser, one of the two editors-in-chief of *microLife*, the journal of the European Academy of Microbiology (EAM); and Jessica Thompson, who had just joined the editorial team of *Nature Microbiology*. It was an exceptionally well-attended and lively session, notwithstanding the fact that it partly overlapped with the lunch break on Thursday. Second, Jörg Vogel and Carmen Buchrieser chaired a panel discussion focused on ‘The importance of microbiology and infectious disease research in the year 2023’ (Fig. 9). The three invited participants included Eliora Ron, EAM Secretary General, and professor of microbiology in Israel, which is another country with a very strong microbiology programme, as well as Seyed E. Hasnain and Feng Shao, who hail from and work in India and China, respectively. The latter two countries together represent almost a third of the world’s population, but it was interesting to learn about their efforts to develop a structured approach to promoting microbiology as a strong discipline in its own right.

## Summary and outlook

**Figure 10.**
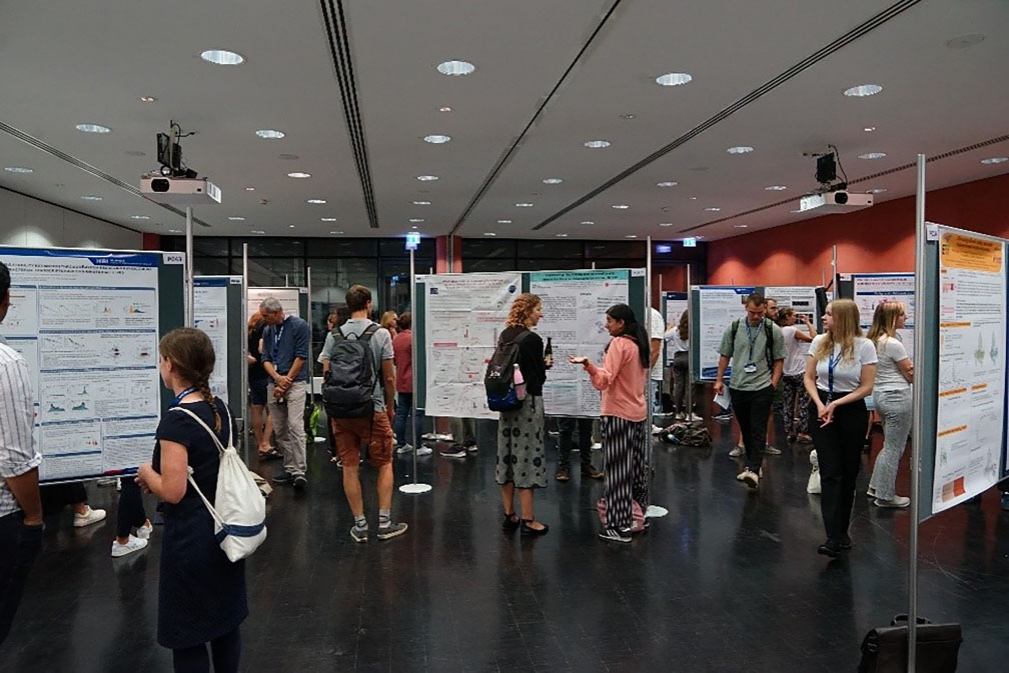
Poster session on Wednesday night.

**Figure 11.**
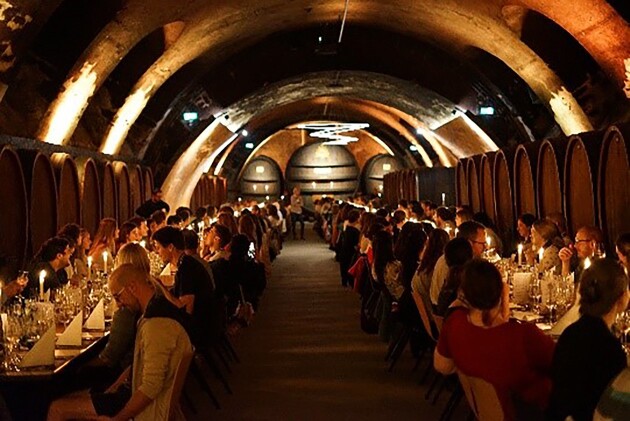

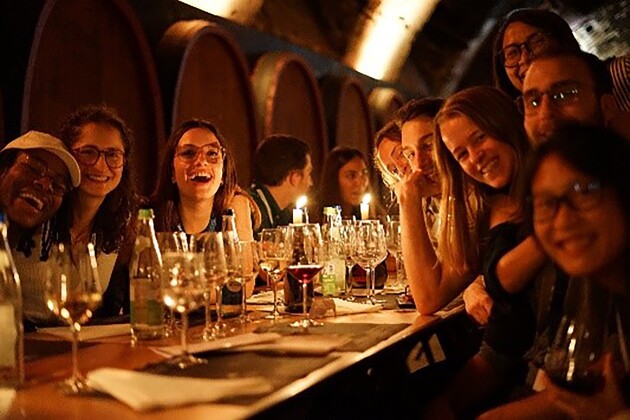
Impressions from the conference dinner at the Staatlicher Hofkeller, located under the Würzburger Residence (a world heritage place), on Thursday night.

These three September days will doubtlessly be remembered for the excellent talks and a general feeling of excitement shared by the organizers, speakers, and attendees of Microbiology 2023. The atrium of the IMIB was buzzing with conversations and scientific discussions during the light dinner and poster session on Wednesday night (Fig. 10). As ever, the Staatlicher Hofkeller, located in the cellars under the Würzburger Residence (a world heritage place), provided a great venue for the conference dinner on Thursday night (Fig. 11). Almost 900 years of history make this place one the oldest wineries in the world.

The organizers are indebted to a great local organization team, which included Hilde Merkert, Monika Schraut, and Mona Alzheimer from the IMIB and Stefanie Ziegler, Tobias Kerrinnes, Britta Grigull, Tim Schnyder, and Luisa Macharowsky from the Würzburg Helmholtz Institute. Thanks to them, the conference went very smoothly and nobody got lost despite the fact that by pure coincidence, the conference venue happened to be surrounded by no fewer than five construction sites in September 2023. These sites included a deep construction pit for the new Helmholtz Institute building right opposite the IMIB. We would also like to acknowledge generous financial support by the Leopoldina, the Alfried Krupp von Bohlen und Halbach-Stiftung as well as intramural funds of the Helmholtz Institute for RNA-based Infection Research.

**Figure 12.**
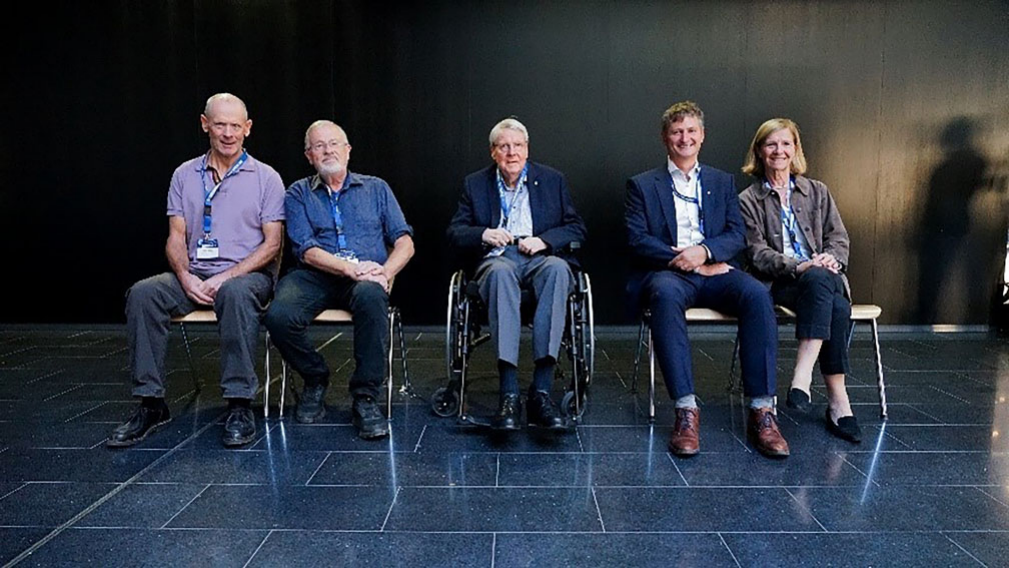

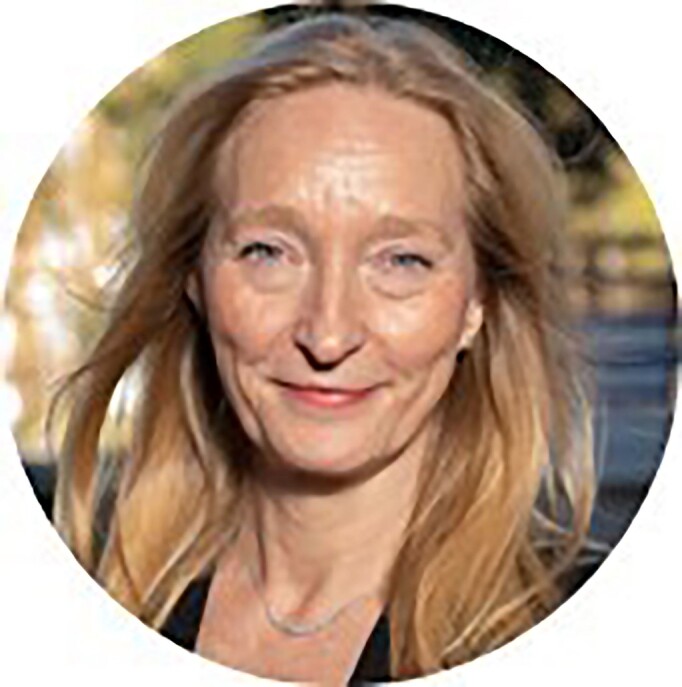
The organizers relaxing on the last day after concluding that the conference had gone well. Lead organizer Birgitta Henriques-Normark for the next Interacademy conference to be held in Sweden pictured on the right.

As Microbiology 2023 ended on Friday afternoon, there was great enthusiasm for the idea of keeping this successful conference format, with the aim to come together and put the spotlight on the latest developments and emerging new concepts in the quest to fathom the intricate lifestyles and molecular compositions of microbes. In this regard, the present organizers were glad to learn that the Swedish national academy, will take the lead in planning the next such Interacademy conference in 2026. While the venue in Sweden is yet to be agreed upon, Birgitta Henriques Normark will serve as the lead organizer (Fig. 12). As a professor of Clinical Microbiology at the Karolinska Institute and the head physician of the associated University Hospital, Henriques Normark is a well-established name in European microbiology. Moreover, having recently been elected President of the Royal Swedish Academy of Sciences, she has a natural interest in partnering with other national academies to serve what is one of the main purposes of these institutions: to nurture a community of scientifically like-minded people and highlight the latest developments in science.
